# *Hermetia illucens* oil vs. hydrogenated palm fat in dairy cow nutrition: effects on digestive parameters, oxidative stress, and milk production performance

**DOI:** 10.1186/s40104-025-01222-9

**Published:** 2025-06-26

**Authors:** Lara Rastello, Laura Gasco, Mauro Coppa, Mathieu Gerbelle, Stefania Colombini, Marco Battelli, Paola Badino, Luca Vernetti-Prot, Pablo G. Toral, Alberto Brugiapaglia, Giulia Gardini, Vanda Malfatto, Isabelle Constant, Alessandro Galli, Christophe Trespeuch, Manuela Renna

**Affiliations:** 1 Department of Veterinary Sciences, University of Turin, Largo P. Braccini 2, Grugliasco, TO 10095 Italy; 2 Department of Agricultural, Forest and Food Sciences, University of Turin, Largo P. Braccini 2, Grugliasco, TO 10095 Italy; 3 Institut Agricole Régional, Regione La Rochère 1/A, Aosta, AO 11100 Italy; 4 Department of Agricultural and Environmental Sciences - Production, Landscape, Agroenergy, University of Milan, Via Celoria 2, Milan, MI 20133 Italy; 5 Instituto de Ganadería de Montaña (CSIC-ULE), Finca Marzanas S/N, Grulleros, 24346 Spain; 6 University of Clermont Auvergne, INRAE, VetAgro Sup, UMR Herbivores, Saint-Genès Champanelle, 63122 France; 7 Ferrero Mangimi SpA, Via Fornace 15, Farigliano, CN Italy; 8 Mutatec, Chemin du Mitan, Cavaillon, 84300 France

**Keywords:** Alternative energy source, Antioxidant capacity, Apparent total-tract digestibility, Black soldier fly, Residual feed intake, Ruminant nutrition

## Abstract

**Background:**

Scant information is currently available on the use of insect oils in ruminant diets. Insect oils could be used as alternatives to certain conventional plant lipid sources that are considered no longer sustainable. This trial aims at evaluating the effects of the dietary inclusion of *Hermetia illucens* oil (HIO) vs. hydrogenated palm fat (HPF) on digestive parameters, oxidative stress, and milk production performance of dairy cows.

**Results:**

Twenty-six Valdostana Red Pied cows were randomly divided into two groups and fed with hay ad libitum and a concentrate containing 3% (as fed) of either HPF or HIO. The trial lasted 50 d, including two weeks of diet adaptation. Individual feed intake and milk yield were monitored three and four times a week, respectively. Fecal samples were collected at the end (d 50) of the trial to determine total-tract nutrients apparent digestibility. Individual blood samples were collected to evaluate blood plasma metabolites (d 0 and d 50) and oxidative stress parameters (d 0, d 26 and d 50). Milk samples were collected at d 0, d 14, d 26, d 38 and d 50 for chemical composition analysis. Feed efficiency was estimated through feed conversion ratio and residual feed intake (RFI). Data were analyzed by SAS software using a mixed model. The diet had no effect on nutrients intake and apparent total-tract digestibility. However, the dietary inclusion of HIO led to higher milk production (+ 0.82 kg/cow/d; *P* < 0.05) and slightly lower RFI (−0.008; *P* < 0.001) when compared to the HPF diet. Milk composition and the nutritional metabolic status of the cows remained unaffected by diet. Serum antioxidant capacity was comparable between the two groups, while lower derivatives of reactive oxygen metabolite concentrations were observed in the HIO-fed cows when compared to the HPF-fed ones (−37.13 Carratelli Units; *P* < 0.001).

**Conclusions:**

The dietary inclusion of HIO instead of HPF did not negatively affect feed palatability and total-tract apparent digestibility of nutrients in dairy cows. Furthermore, it increased feed efficiency by supporting a higher milk production together with an improved antioxidant status. The results suggest that HIO could be an eligible option as an innovative energy source for dairy cows.

## Background

Palm fat is one of the most used energy sources in ruminant nutrition [1]. Medium-chain saturated fatty acids (SFA) are the predominant lipid fraction in palm fat, with palmitic acid (C16:0) being the most abundant individual fatty acid (FA) [2]. Medium-chain SFA rapidly undergo β-oxidation inside the liver, being quickly metabolized and serving as an immediate energy source for the animal [3]. The addition of oils or fats rich in medium-chain SFA in the diet of dairy cows is mainly accomplished to meet their energy demand and to support their milk production, especially in the period around peak of lactation, when the cows experience a negative energy balance [4]. Research has shown that incorporating palm fat in the diet of lactating animals can determine positive outcomes, including increased animal productive performance and health [5]. However, palm oil production has become highly controversial [6]. Even if efforts have been recently made by producers to make palm oil production more sustainable, the latter is associated with several negative social and environmental impacts, including threats to small-holder livelihoods, massive deforestation, loss of biodiversity, soil and water pollution, and contribution to climate change [6]. Therefore, ruminant nutritionists worldwide are striving to find more sustainable solutions.

In the last years, increasing interest has been addressed by feed companies, nutritionists and researchers, on the possibility of using insect-derived products in the nutrition of farmed animals [7]. Insect meals are currently banned in ruminant nutrition in many high-income countries [8]. In the European Union (EU), such ban regards the use of processed animal proteins and is aimed at preventing and eradicating the potential risk of Bovine Spongiform Encephalopathy (Regulation EU No 2001/999) [9, 10]. However, insect oils, usually extracted from the larvae to allow the production of high-protein insect meals [11], are not subjected to the above-mentioned restrictions and are gaining attention for their potential application as sustainable energy sources in ruminant diets.

Black soldier fly (*Hermetia illucens* L.; HI) is one of the most promising insect species used for feed purposes worldwide. The reasons are many, mostly deriving from its high versatility. Indeed, HI can be produced all over the world and can be reared on a highly diversified spectrum of organic waste [12]. The high adaptability of this insect species enhances the actualization of Circular Economy models by converting waste into valuable nutrients [13]. As a counterbalance, HI industry, being younger than that of plant-based feed ingredients, lacks standardized practices and it is currently characterised by small-scale manufacturing as well as high and not yet competitive market price of derived products [14, 15].

The HI oil (HIO) and palm fat share a comparable FA profile, which makes the former an interesting potential alternative to the latter. Similarly to palm fat, HIO mainly contains medium-chain SFA, but with a clear prevalence of lauric (C12:0) over palmitic acid [16]. Lauric acid is known to possess antioxidant, antimicrobial, antifungal, and antiviral properties [17], which make it attractive also from an animal health perspective. Additionally, the presence of other bioactive compounds in HIO (namely eicosanoids, oxylipins, and isoprenoids) [18] suggests that its dietary inclusion in ruminant diets may help to mitigate oxidative stress and inflammation, by enhancing the antioxidant defense system of the animals [19].

Despite great potential for application, the current use of insect-derived products in ruminant nutrition is very limited and numerous knowledge gaps need to be covered. To the best of our knowledge, only one in vivo study has been published so far on the effects of dietary HIO supplementation on milk production, milk quality and biochemical blood profile of dairy cows [20]. With the current study, we aimed at evaluating the effects of using HIO instead of hydrogenated palm fat (HPF) in the diet of dairy cows, with a focus on voluntary feed intake, apparent total-tract digestibility of nutrients, plasma nutritional metabolites, oxidative stress status, milk production performance, and feed efficiency.

## Methods

### Experimental design, animals and diets

The trial was conducted at the experimental farm of the Institute Agricole Régional, located in Montfleury (Aosta, NW Italy, latitude: 45°43′54′′ N, longitude: 7°17′56′′ E, altitude: 577 m a.s.l). Twenty-six Valdostana Red Pied cows were selected from a herd of 33 lactating cows and randomly allocated to two groups (13 cows per group), balanced for body weight (BW; 556 ± 49.5 kg), parity (3.0 ± 1.91), stage of lactation (68 ± 27.5 d in milk), milk yield (22.0 ± 3.64 kg/cow/d) and milk main constituents (fat: 34.5 ± 0.48 g/kg, protein: 31.4 ± 0.30 g/kg, lactose: 47.2 ± 0.15 g/kg, casein: 24.8 ± 0.24 g/kg, and urea: 18.2 ± 3.50 mg/dL). Before starting the trial, all the selected cows received a common diet characterized by an average forage to concentrate (F:C) ratio equal to 70:30, on a DM basis. This diet was composed of mixed hay (75% of first cut hay and 25% of third cut hay) and a concentrate which was similar to the concentrates used during the experimental period, but without any lipid source included.

The experiment lasted 50 d, including two weeks of diet adaptation at the beginning of the trial. During the experimental period, the cows were individually fed with diets characterized by an average F:C ratio equal to 70:30, on a DM basis (observed variations, based on voluntary feed intake of hay, ranged from 68:32 to 72:28). The experimental diets were formulated to meet the nutritional requirements of the animals [21]. The roughage part of the diet was the same as that of the pre-experimental period for both groups of cows and was administered ad libitum. The pelleted concentrates administered to the animals were also identical for the two experimental groups (Table 1), with the only exception of the main lipid source, which was HPF for the control group (HPF group) and HIO for the other group (HIO group). The lipid source was included within the concentrate at 3% as fed. The concentrates (fixed total daily amount equal to 4.8 kg/cow, on a DM basis) were automatically administered to each cow in equal quantities at 04:45 h, 07:15 h, 10:10 h, 15:30 h, 19:30 h and 23:30 h. The animals were individually housed indoors in tie-stall conditions and had free access to clean and fresh water and salt blocks (SOLSEL^®^ Natural, K + S Minerals and Agriculture GmbH, Kassel, Germany) throughout the entire trial. The HIO was produced by Mutatec (Cavaillon, France) and was obtained from HI larvae reared on a vegetable substrate. The HI larvae were slaughtered by using the steam bleaching procedure and then they were subjected to an initial drying step (95 °C in a vibrator-dryer) to remove the moisture content. Finally, the dehydrated larvae were pressed and left to naturally decant to obtain HI meal and oil without the use of any solvent. Hydrogenated palm fat was supplied by Ferrero Mangimi SpA (Farigliano, Italy). The main FAs in HIO were lauric, linoleic (C18:2 n-6), palmitic and oleic (C18:1 *c*9) acids (36%, 15%, 12%, and 11% of total detected FA, respectively), while the HPF mainly contained palmitic, stearic and oleic acids (84%, 10%, and 5%, respectively).

**Table 1:** Ingredient composition (g/100 g DM) of the two experimental concentrates containing hydrogenated palm fat (HPF) or *Hermetia illucens* oil (HIO)

Ingredients	Dietary treatment	
	**HPF**	**HIO**
Corn meal	29.6	29.6
Dehulled sunflower meal (36% CP)	17.0	17.0
Wheat bran	16.0	16.0
Wheat middlings	10.7	10.7
Barley meal	7.9	7.9
Soybean meal (44% CP)	5.9	5.9
Palm hydrogenated fat	3.4	-
*Hermetia illucens* oil	-	3.4
Pitted carobs	3.2	3.2
Calcium carbonate (CaCO_3_)	2.3	2.3
Cane molasses	1.2	1.2
Sodium chloride (NaCl)	1.1	1.1
Dolomite (CaMg(CO_3_)_2_)	1.1	1.1
Sodium bicarbonate	0.6	0.6
Vitamin mixture^a^	0.3	0.3

*CP* Crude protein

^a^Containing per 100 g of product: Vitamin A, 20,000 IU; Vitamin D_3_, 1,600 IU; Vitamin E, 40 mg

### Sampling and analysis

#### Chemical analysis of feeds

Hays and concentrates were sampled at the beginning (d 0, the day before starting the administration of the experimental diets), middle (d 25) and end (d 50) of the trial to be analyzed for their proximate composition. All the feedstuffs were ground with a cutting mill to pass a 1-mm screen sieve (Pulverisette 15-Fritsch GmbH, Idar-Oberstein, Germany). AOAC International procedures [22] were used to determine DM (method no. 930.15), ash (method no. 942.05), crude protein (CP) (method no. 984.13), acid detergent fiber (ADFom, corrected for residual ash) and acid detergent lignin (ADL) (method no. 973.18). Method no. 2003.05 of AOAC International [23] was used to determine the ether extract (EE) content. Neutral detergent fiber (aNDFom) was determined according to Mertens [24] by adding α-amylase (Merck, Darmstadt, Germany) and sodium sulfite (Merck, Darmstadt, Germany), and correcting for residual ash content. Rumen degradable protein (RDP) was analyzed according to Licitra et al. [25] and expressed as g/100 g CP, while rumen undegradable protein (RUP) was calculated as 100 – RDP. Non-structural carbohydrates (NSC) were calculated as 100 – (CP + EE + ash + aNDFom). The energetic value of the feedstuffs was estimated following NASEM [26].

#### Feed and nutrients intake

Individual feed intake was recorded thrice a week, from d 0 until d 50, separately for hay and concentrate, by weighing the administered feed and the refusals.

Individual daily nutrients intake (ash, CP, RDP, RUP, EE, NSC, aNDFom, ADFom, ADL), expressed as kg/cow, was estimated considering the average daily DM intake and the analytically determined composition of the hays and concentrates used in the trial.

#### Apparent total-tract digestibility

Individual fecal samples were collected at d 50 using the spot sampling method, approximately 6 h after the morning feeding and directly from the rectal ampoule, according to Cavallini et al. [27]. Feces were then oven-dried at 55 °C until they achieved constant weight and subsequently ground to pass a 1-mm sieve (Pulverisette 19, Fritsch GmbH, Idar-Oberstein, Germany) to be chemically analyzed. Chemical analyses of individual fecal samples for DM, ash and fiber fractions were performed as previously described for feed samples. The fecal EE content was also determined as previously described, but with preliminary acid hydrolysis (EC Regulation No 152/2009) [28].

The undigested NDF (uNDF) of feedstuffs and feces was used as an internal marker to assess the total-tract digestibility of DM, organic matter (OM), aNDFom, and EE (DMd, OMd, NDFd, and EEd, respectively). The uNDF was estimated after 288 h in vitro incubation using Daisy^II^ Incubator jars (Ankom Technology, Macedon, NY, USA). For each sample of the two diets (hays and concentrates), eight replicates (two for each jar) were prepared with 0.5 g of sample in F57 Ankom bags [29]. Differently, for the fecal samples, four replicates were prepared (one bag for each jar). The bags were pre-treated according to Battelli et al. [30]. Each jar contained one bag with alfalfa hay as internal standard feed and two empty bags (blanks) as internal test of the lab to evaluate the goodness of the in vitro run. Rumen fluid was collected 2 h after the morning feeding from three rumen-cannulated dry Italian Holstein cows housed in the experimental farm “Cascina Baciocca”, of the Milan University Farms Functional Centre (Cfaa) located in Cornaredo (Milan, Italy). The cows were fed a diet composed of 66% grass hay and 34% concentrate based on corn meal, soybean meal, wheat bran and min-vit. Rumen fluid was immediately filtered with four layers of cheesecloth and flushed with CO_2_ in a 39 °C prewarmed flask. Rumen fluid was then mixed with the buffer solution prepared as indicated by the Ankom Daisy^II^ protocol [29]. The rumen fluid and the buffer solution of each jar were then replaced at 144 h of incubation, to restore the optimal ruminal microbial activities. After the 288-h incubation, the samples were analyzed for their uNDF contents.

For both the feces and the diets, the DMd (1) and nutrient digestibility (2) were assessed according to the following equations:

1 $$\text{DMd }= 100 - (100 \times \frac{\text{\%uNDF in diets}}{\text{\%uNDF in feces}})$$

2 $$\text{Nutrient digestibility }= 100 - (100 \times \frac{\text{\%uNDF in diets}}{\text{\%uNDF in feces}} \times \frac{\text{\%Fecal nutrient content}}{\text{\%Diet nutrient content}})$$

#### Plasma parameters

As far as plasma metabolites are concerned, individual blood sampling occurred, before the morning milking and feeding, from the jugular vein at d 0 and d 50, by using tubes containing EDTA (2.1 mg/mL). Immediately after sampling, the EDTA tubes were stored in a refrigerated bag and transported to a laboratory for plasma separation, which occurred by centrifugation at 2,000 × *g* for 15 min at 4 °C. Then, the samples were stored at –20 °C until analyses. Plasma parameters were assayed using colorimetric enzymatic reactions with a multiparameter analyzer (Arena 20XT; Thermo Scientific, Vaanta, Finland) to determine concentrations of non-esterified FA (NEFA) (Wako NEFA HR2 kit, Oxoid, Dardilly, France), cholesterol (product code 981813, Thermo Scientific; Waltham, MA, USA), glucose (Glucose RTU kit BioMérieux, Lyon, France), triglycerides (product code 981786, Thermo Scientific; Waltham, MA, USA), urea (product code 981954, Thermo Scientific; Waltham, MA, USA), and β-hydroxybutyrate (BHB) according to the method described by Brashear and Cook [31].

#### Oxidative stress

Blood samples for oxidative stress evaluation were collected at d 0, d 26, and d 50 by using 10 mL vacutainers without anticoagulant (Venosafe®, Terumo Corporation, Tokyo, Japan) before the morning milking and feeding. Blood was then centrifuged at 2500 × *g* for 10 min at room temperature and stored at −80 °C until analysis. Serum antioxidant capacity (SAC) was evaluated using the OXY-Adsorbent test (Diacron International, Grosseto, Italy), that quantifies the resistance of the serum to oxidation induced by a solution of hypochlorous acid (HClO). The analysis was performed according to the indications reported by Pirro et al. [32]. Results were expressed as µmol of HClO per mL of sample.

Samples were then tested to determine the derivatives of reactive oxygen metabolites (d-ROMs), which are an indicator of oxidative stress due to the presence of free radicals in the blood. This parameter was evaluated using the d-ROMs test (Diacron International Srl, Grosseto, Italy) according to the instructions provided by the manufacturer and the protocol described by Sauerwein et al. [33]. Absorbance was measured by photometric reading at 505 nm and results were expressed in Carratelli Units (UCARR; 1 UCARR corresponds to 0.08 mg H_2_O_2_/dL) using a human standard serum as calibrator after blank subtraction.

#### Milk yield and main constituents

The cows were milked twice a day (at 06:00 h and 18:00 h) by using an automatic milking machine (DeLaval, DelPro “MU486”, Milan, Italy). Individual milk yield was recorded 4 d a week during the whole trial. Proportional composite samples of the morning and afternoon milkings (according to milk production recorded per milking) were collected from each cow at d 0, and then at d 14, d 26, d 38, and d 50. A composite milk sample of 50 mL was analyzed for fat, protein, casein, lactose, and urea contents (MilkoScan FT 6000, Foss Electric, Hillerød, Denmark), and for somatic cell count (SCC) (Fossomatic FC, Foss Electric, Hillerød, Denmark). The energy corrected milk (ECM) was calculated following INRA [21], while the fat and protein corrected milk (FPCM) was calculated using the formula proposed by Yan et al. [34].

#### Feed efficiency predictors

The individual body weight (BW) of the cows was used for feed efficiency calculations. It was measured for two consecutive days immediately before d 0, and for two consecutive days immediately after d 50, always before the morning meal. Two feed efficiency metrics were calculated: (i) the FCR, as the ratio between dry matter intake (DMI) and ECM; and (ii) the RFI by using a regression model, as detailed in the following section of the manuscript.

### Statistical analyses

The SAS software package (version 9.4; SAS Institute Inc., Cary, NC, USA) was used for the statistical analysis of data.

Data about DMI, nutrients intake, milk yield, ECM, FPCM, milk main constituents and oxidative stress parameters were analyzed using a MIXED model for repeated measures over time, including the fixed effects of diet and interaction between diet and time (D × T), the animal as a random effect, and the initial measure of each parameter at d 0 as a covariate. To achieve normal distribution, the SCC data was log_10_ transformed prior to being statistically analyzed, but results are presented as non-transformed data. The same model without the repeated factor was used to analyze plasma parameters; in the case of apparent total-tract digestibility, the covariate period was also removed from the model [35]. Results are reported as least squares means and standard error of the mean (SEM).

Statistical analysis on individual concentrate intake was not performed, since the experimental concentrates were given in the same amount and always eaten by the animals with no refusals.

The RFI values were estimated for the 26 cows as residuals of the following regression model, using the GLM procedure:

$$\text{DMI }=\upmu +\text{ a }\times \text{ ECM }+\text{ b }\times \text{ MBW }+\text{ c }\times \text{ BWC }+\text{ RFI}$$

where DMI represents the mean DMI of data collected individually over a 50-d period (kg/d); μ is the intercept; ECM is the energy-corrected milk yield (L/d); MBW is the mean metabolic BW (BW^0.75^; kg); BWC is the BW change over the period (kg); RFI is the residual; a, b, and c are the regression coefficients. In an initial analysis, days post-partum were also included in the regression model, but since this variable was not significant (*P* = 0.404), it was removed from the final model.

Significance was declared at *P*-values ≤ 0.05, while 0.05 < *P*-values ≤ 0.10 were interpreted as a trend toward significance.

## Results

### Feed intake and apparent total-tract digestibility

The voluntary total DMI and the nutrients intake are reported in Table 2. The lipid sources represented approximately 0.9% of total DMI. The dietary treatment did not affect the total DMI of the cows, nor their intake of hay or concentrate (*P* > 0.05). No differences were also observed when analyzing the individual intake of ash, CP, RDP, RUP, EE, NSC, ADFom and ADL between the experimental groups (*P* > 0.05). The HIO-fed cows showed lower ingestion of aNDFom when compared to the HPF-fed ones (−0.23 kg/cow/d; *P* < 0.05). The D × T interaction was not significant for all the considered parameters.

**Table 2:** Dry matter and main nutrient intakes (kg/cow/d, unless otherwise stated) of dairy cows fed diets containing hydrogenated palm fat (HPF) or *Hermetia illucens* oil (HIO)^a^

Main nutrients	Experimental diets		SEM	*P*-value		
	**HPF**	**HIO**		**D**	**T**	**D × T**
	**(*n*** **= 13)**	**(*n*** **= 13)**				
Total DM intake	15.7	15.4	0.24	0.083	0.014	0.382
DM intake from hay	11.1	10.8	0.24	0.100	0.014	0.382
DM intake from concentrate^b^	4.8	4.8	-	-	-	-
Ash	1.44	1.43	0.023	0.493	< 0.001	0.330
CP	2.02	1.99	0.022	0.079	< 0.001	0.446
RDP	1.11	1.09	0.013	0.054	0.014	0.382
RUP	0.91	0.90	0.009	0.119	0.014	0.382
EE	0.50	0.49	0.005	0.082	< 0.001	0.120
NSC^c^	4.26	4.24	0.052	0.511	0.014	0.382
aNDFom	7.74	7.51	0.137	0.020	< 0.001	0.353
ADFom	4.63	4.55	0.087	0.203	< 0.001	0.376
ADL	0.62	0.61	0.010	0.057	0.001	0.308
NE_L_,Mcal/cow/d	21.6	21.2	0.280	0.061	0.014	0.382

*n* Number of samples, *SEM* Standard error of the mean, *D* Ddiet, *T* Sampling time, *D* × *T* Interaction between diet and sampling time, *DM* Dry matter, *CP* Crude protein, *RDP* Rumen degradable protein, *RUP* Rumen undegradable protein, *EE* Ether extract, *NSC* Non-structural carbohydrates, *aNDFom* Neutral detergent fiber, *ADFom* Acid detergent fiber, *ADL* Acid detergent lignin, *NEL* Net energy for lactation

^a^Total number of measurements for each group equals to 260 (13 cows × 20 sampling days)

^b^Statistical analysis was not performed, since all the concentrates were given in the same amount in the two diets and always eaten by the animals with no refusals

^c^Calculated as: 100 − (CP + EE + Ash + aNDFom)

No significant differences were observed in terms of DMd, OMd and EEd between the experimental groups, while the NDFd tended to be lower in the HIO-fed cows when compared to HPF-fed ones (−2.1%; *P* = 0.060; Table 3).

**Table 3:** Apparent total-tract digestibility (as % of dry matter intake) in dairy cows fed diets containing hydrogenated palm fat (HPF) or *Hermetia illucens* oil (HIO)

Item	Dietary treatment		SEM	*P-*value
	**HPF**	**HIO**		
	**(*n* = 13)**	**(*n* = 13)**		
DMd	72.3	73.4	0.53	0.145
OMd	74.5	73.5	0.52	0.175
NDFd	60.9	58.8	0.75	0.060
EEd	83.6	83.3	0.44	0.583

*n* number of samples, *SEM *Standard error of the mean, *DMd* Apparent total tract dry matter digestibility, *OMd* Aapparent total tract organic matter digestibility, *NDFd* Apparent total tract neutral detergent fiber digestibility, *EEd* Apparent total tract ether extract digestibility

### Plasma parameters and oxidative stress

No differences were observed for plasma parameters (i.e., NEFA, cholesterol, glucose, triglycerides, urea, and BHB) when comparing the HPF-fed and the HIO-fed cows (Table 4).

**Table 4:** Concentrations of plasma metabolites in dairy cows fed diets containing hydrogenated palm fat (HPF) or *Hermetia illucens* oil (HIO)

Item	Dietary treatment		SEM	*P-*value
	**HPF**	**HIO**		
	**(*n* = 13)**	**(*n* = 13)**		
NEFA, mmol/L	0.22	0.27	0.030	0.260
Cholesterol, g/L	2.65	2.79	0.083	0.240
Glucose, g/L	0.63	0.65	0.011	0.174
Triglycerides, g/L	0.15	0.16	0.007	0.187
Urea, g/L	0.17	0.16	0.006	0.744
BHB, mmol/L	0.57	0.64	0.130	0.696

*n* Number of samples, *SEM* Standard error of the mean, *NEFA* Non-esterified fatty acids, *BHB* β-Hydroxybutyrate

The inclusion of HIO in the diet of the cows did not affect SAC values (Table 5). The results show no significant differences between the two experimental groups at all sampling times, with an overall mean of 347.1 µmol HClO/mL for the HPF group and 339.9 µmol HClO/mL for the HIO group (*P* > 0.05). However, the cows fed the HIO diet showed lower d-ROMs concentrations when compared to those fed the HPF diet (−37.13 UCARR; *P* < 0.001). The D × T interaction was not significant for SAC and d-ROMs values.

**Table 5:** Serum antioxidant capacity values (µmol HClO/mL) and serum d-ROMs values (UCARR) in dairy cows fed diets containing hydrogenated palm fat (HPF) or *Hermetia illucens* oil (HIO)^a^

Item	Dietary treatment		SEM	*P*-value		
	**HPF**	**HIO**		**D**	**T**	**D × T**
	**(*n* = 13)**	**(*n* = 13)**				
SAC	347.1	339.9	11.09	0.556	0.012	0.464
d-ROM	110.84	73.71	8.322	< 0.001	0.487	0.157

*n* Number of samples, *SEM* Standard error of the mean, *D* Diet, *T* Sampling time, *D* × *T* Interaction between diet and sampling time, *SAC* Serum antioxidant capacity, *d-ROM* Derivatives reactive oxygen metabolites

^a^Total number of measurements for each group equals to 78 (13 cows × 3 sampling days)

### Milk yield and main constituents

The HIO-fed cows showed significantly higher individual milk yield (+ 0.82 kg/cow/d; *P* < 0.01), ECM and FPCM (+ 0.74 kg/cow/d and + 0.72 kg/cow/d, respectively; *P* < 0.05) when compared to the HPF-fed cows (Table 6). No significant differences were observed between the two groups in terms of milk main constituents (i.e., fat, protein, casein, and lactose contents and yields), and milk SCC. The urea content of milk tended to be higher in the HIO-fed cows when compared to the HPF-fed ones (+ 0.98 mg/dL; *P* = 0.095). The D × T interaction was not significant for all the considered parameters.

**Table 6:** Milk yield and composition of dairy cows fed diets containing hydrogenated palm fat (HPF) or *Hermetia illucens* oil (HIO)^a^

Item	Dietary treatment		SEM	*P*-value		
	**HPF**	**HIO**		**D**	**T**	**D × T**
	**(*n* = 13)**	**(*n* = 13)**				
Milk yield, kg/cow/d	19.5	20.3	0.431	0.008	0.112	0.981
ECM, kg/cow/d	17.3	18.1	0.495	0.038	0.037	0.359
FPCM, kg/cow/d	17.0	17.7	0.481	0.037	0.026	0.391
Milk composition						
Fat, g/kg	37.8	38.9	1.16	0.218	0.099	0.118
Protein, g/kg	30.4	30.7	0.43	0.468	0.007	0.940
Casein, g/kg	24.3	24.2	0.36	0.609	0.040	0.966
Lactose, g/kg	47.8	47.6	0.27	0.310	0.548	0.443
SCC, n°/mL × 10^3^	25.9	20.8	0.80	0.125	0.591	0.245
Urea, mg/dL	18.8	19.7	0.78	0.095	< 0.001	0.348
Component yield, g/cow/d						
Fat	754	775	28.1	0.277	0.452	0.244
Protein	606	608	11.8	0.829	0.005	0.997
Casein	481	483	9.6	0.733	0.013	0.999
Lactose	953	956	22.5	0.833	0.451	0.926

*n* Nnumber of samples, *SEM* Standard error of the mean, *D* Diet, *T* Sampling time, *D* × *T* Interaction between diet and sampling time, *ECM* Energy corrected milk, *FPCM* Fat-protein corrected milk, *SCC* Somatic cell count

^a^Total number of measurements for milk yield for each group equal to 390 (13 cows × 30 sampling days), while those for milk composition and component yield for each group equal to 52 (13 cows × 4 times of sampling)

### Feed efficiency predictors

No differences in the BW or BWC of the animals between the two experimental groups were observed (*P* > 0.05; Table 7). The inclusion of HIO positively affected the RFI of the cows compared to HPF, although differences between groups were small (−0.008 for the latter group; *P* < 0.001; Table 7). On the contrary, the FCR remained unaffected by diet (*P* > 0.05).

**Table 7:** Body weight (kg), body weight change (kg/period) and feed efficiency predictors of dairy cows fed diets containing hydrogenated palm fat (HPF) or *Hermetia illucens* oil (HIO)

Item	HPF	HIO	SEM	*P*-value
	**(*n* = 13)**	**(*n* = 13)**		
BW	542.1	545.7	4.48	0.576
BWC	−10.19	−14.10	4.680	0.561
RFI	0.004	−0.004	0.0018	< 0.001
FCR	0.81	0.81	0.022	0.820

*n* Number of samples, *SEM* Standard error of the mean, *BW* Body weight, *BWC* Body weight change over the trial period, *RFI* Residual feed intake, *FCR* Feed conversion ratio

## Discussion

### Feed intake and apparent total-tract digestibility

This study evaluates the effects of the inclusion of two lipid sources with a different medium-chain SFA profile as feed ingredients in the diet of lactating cows. One critical aspect to be considered when lipid sources are used is the EE content of the diet since lipids can decrease DMI and digestibility due to possible lower palatability and negative effects on cellulolytic bacteria [1]. However, in the present study the lipid content of the diet was 3.03 g/100 g DM; such value is much lower than the threshold value of 6% of the total dietary DM [26] which has classically been suggested to have negative effects on DMI and total-tract digestibility of nutrients [36].

Another critical aspect when introducing new feed ingredients is their palatability, as low palatability can further decrease DMI. In the current study, the diet containing HIO showed comparable palatability to that containing HPF, as evidenced by the lack of significant effects on DM and nutrient intakes. This finding suggests that HIO may be a palatable alternative to traditional plant-derived and mostly saturated fats, such as palm oil or coconut oil, in cattle diets, which could have positive implications for feed formulation and animal performance. Lauric acid is the most abundant individual FA in HIO, comprising around 40 g/100 g of total FA [37]. In the current study, the amount of lauric acid in HIO was equal to 36 g/100 g of total FA, and the total supply of HIO as fed was equal to 162 g/cow/d, corresponding to around 58 g of lauric acid. Based on the actual lauric acid intake levels, we did not expect a decrease in DMI by the HIO-fed cows when compared to HPF-fed ones. Indeed, Zhang et al. [38] found no significant variations in the DMI of dairy cows when supplementing a total mixed ration (TMR) (50:50 F:C ratio and 3.2 g EE/100 g DM) with 100 and 200 g lauric acid/cow/d, when compared to the unsupplemented control diet. On the contrary, different outputs are documented in published literature with higher lauric acid intakes. For example, lauric acid intakes equal to 404 and 543 g/cow/d in a TMR diet drastically reduced DMI (−24% when compared to a control diet not containing lauric acid) in lactating Holstein cows [39]. These findings underscore and confirm the critical need to consider dosage and threshold values when assessing the impact of lauric acid, and by extension, of HIO on feed palatability and DMI. Similar results in terms of DMI reduction were obtained when coconut oil, which has a FA profile which closely resembles that of HIO, was fed to lactating cows [40]. In accordance with the results obtained by Zhang et al. [38], assuming a threshold level of lauric acid intake of 200 g/cow/d, a dietary HIO inclusion of about 3.7% DM seems feasible.

Overall, in the current study, DM digestibility was also unaffected by the lipid source. By means of an in vitro study, and using cannulated ewes as inoculum donors, Hervás et al. [41] compared the in vitro DM disappearance of a TMR (55:45 F:C ratio, containing dehydrated alfalfa as roughage source) supplemented with HIO or with a traditional vegetable oil (soybean oil), with both the ingredients tested at 20 g/kg DM of dosage. After 16 h in vitro incubation, these authors observed no difference in DM disappearance between HIO and soybean oil as compared to the control treatment (no oil supplementation). Similarly, adding HIO at 5% DM to two diets characterized by contrasting F:C ratios (70:30 and 30:70) and containing 3.86 and 5.19 g/100 g DM of crude fat respectively, Jayanegara et al. [42] observed no significant impairments of DMd and OMd when compared to the respective unsupplemented control diets.

In the current study, the difference in FA composition between the two experimental diets probably affected the NDFd, which tended to be slightly lower for the HIO-fed than the HPF-fed cows (*P* = 0.060), suggesting a potential inhibitory effect of HIO on rumen fibrolytic bacteria or protozoa. Available findings about dietary FA profile and its metabolic and physiological effects on dairy cows suggested that not all long-chain FA negatively affect total-tract fiber digestibility [43]. For example, a recent study by Sears et al. [44] indicated that palmitic acid increased in vitro fiber digestibility by 6 percentage units compared to a control diet. Such an increase, which agrees with our NDFd results, was found to be associated with shifts in bacterial FA metabolism, that resulted in modifications of growth and abundance of fiber-degrading bacteria. On the other hand, several studies reported that lauric acid exerts negative effects on fiber digestibility. For example, Vadroňová et al. [45] found that the relative abundance of *Fibrobacter* (fibrolytic bacteria) in ruminal fluid significantly decreased when cows were supplemented with a dietary mixture of capric and lauric acids (50 g of each FA/cow/d added to a silage-based basal diet) compared to a control group supplemented with 100 g of stearic acid. Similarly, Faciola and Broderick [39] reported a linear decrease in NDFd with increasing lauric acid supplementation. Based on their data, a theoretical regression model was derived, predicting a decrease of 0.0286 percentage points in NDFd for each gram of lauric acid intake. Applying this model to the lauric acid intake in our study, we would have expected a decrease of −1.4 percentage points in NDFd, compared to a diet without lauric acid. This expected value is reasonably close to the actual observed reduction of −2.1 percentage points, although towards a diet containing palmitic acid.

The decrease in NDFd observed in our study also aligns with the findings of Yanza et al. [46], who, in a meta-analysis of in vitro and in vivo studies, reported a linear decrease in fiber digestibility with increasing concentrations of medium-chain SFA in ruminant diets. The observed tendency for an inhibition of fiber digestibility, probably imputable to the relatively high content of medium-chain SFA in HIO, could have implications for the energy availability for the animal, as NDF represents a significant portion of the carbohydrate fraction in ruminant diets. However, it is important to highlight that, in the current study, the decrease in NDFd did not negatively affect milk production, as later outlined and discussed.

### Plasma parameters

Plasma parameters are important metabolic health indicators that give information about the energy balance status and the body reserves mobilization of the animals, since they are strictly related to the animals’ diet and nutritional metabolism [47]. In our study, all the values of the tested plasma parameters fell within physiological ranges [48], and no detrimental effects on the metabolic health and the nutritional status of dairy cows were observed when HIO was included in the diet. From published literature, it can be highlighted that only lauric acid intakes higher than that recorded in the current trial may significantly affect the considered plasma parameters. For example, Zhang et al. [38] found that the dietary addition of lauric acid (100, 200 or 300 g/cow/d) linearly increased blood levels of glucose and triglycerides, while an opposite effect was observed for blood NEFA concentrations. However, the same authors showed that the comparison between the unsupplemented control group and the group which received the lowest lauric acid supplementation (100 g/cow/d) showed no statistically significant differences in terms of the above-mentioned blood metabolites. Recently Culbertson et al. [49] also showed that the dietary supplementation of 50 or 150 g/cow/d of glycerol monolaurate, a monoglyceride composed of ester-bonded glycerol and lauric acid, did not affect plasma glucose or NEFA concentrations when compared to an unsupplemented diet composed of corn silage, haylage and concentrate (60:40 F:C ratio and 4 g/100 g DM of crude fat). Our trial was conducted around peak of lactation, a phase characterized by negative energy balance [4]. In this phase, cows are expected to mobilize lipids from their body reserves, increasing the release of NEFA and BHB into blood circulation [4]. Despite a higher milk yield of HIO-fed cows when compared to HPF-fed ones, the milk fat, protein and lactose yields were comparable between the experimental groups. In addition, in feed efficiency estimations, the mean energy balance for HIO- and HPF-fed cows over the experiment was comparable (mean ± SEM; −4.46 ± 2.334 and −4.60 ± 2.776 MJ/d, respectively), which is in agreement with the lack of differences in blood metabolites.

### Oxidative stress

In livestock systems, there are many different stressors that can depress animal performance by inducing them into stressful conditions. Common stressors include lactation, pregnancy, transport, and environmental (e.g., temperature and humidity) changes [50, 51]. With the diet, animals can be supported in strengthening their immune system to fight against the pro-inflammatory mechanisms that normally develop in farming contexts. Nekrasov et al. [20] highlighted for the first time the potential effect of HIO against oxidative stress in ruminants. The supplementation with HIO at 10 and 100 g/cow/d led to an increase in the total amount of water-soluble antioxidants in the blood serum, with the highest level obtained in the group receiving the highest dose of HIO. Similarly, our results revealed an increased resistance to oxidative stress in dairy cows fed with HIO when compared to those fed HPF. Luan et al. [52] recently studied the effects of short- and medium-chain FA triglycerides (C8, C10 and C12) on serum antioxidant capacity and oxidative stress indices in finishing bulls under high-concentrate feeding conditions. Consistently with our findings, these authors reported that the dietary supplementation of glycerol monolaurate at the dose of 60 g/bull/d significantly increased the serum total antioxidant capacity, contemporarily decreasing ROS concentrations, when compared to an unsupplemented control diet. These authors further elucidated the mechanisms behind such effects, showing that medium-chain FA triglycerides can stimulate ghrelin secretion in ruminants. Ghrelin is an endogenous antioxidant that functions as a free radical scavenger and can attenuate oxidative stress levels in different organs [53]. Indeed, in our study, the higher intake of medium chain SFA (particularly lauric acid) with the HIO diet compared to the HPF one might have led to a higher efficiency of ghrelin acylation, which is involved in the regulation of oxidative stress [52] by means of different modes of action (e.g., by increasing antioxidant enzyme activities, by inhibiting cellular apoptosis and ROS formation, and by reducing lipid peroxidation and cytochrome c release) [53]. Also, in monogastric studies several authors showed that the dietary inclusion of HI can exert positive effects by increasing total antioxidant capacity and lowering ROS concentrations in the serum, resulting in higher protection against stressor factors and an overall improvement of animal health [54–57].

HI can synthesize eumelanin pigments throughout all its life cycle. Normally most (around 80%) of these pigments are found on the surface of the insect, bound to chitin, and are available in HI meals, while the remaining 20% is present in the lipid fraction and is mostly bound to lauric acid [57]. Eumelanin pigments exhibit various functional properties (including radical scavenging activities and redox functions as shielding cells from damage caused by free radicals) [58, 59]. The presence of these pigments in HIO may further explain the observed improvement of the oxidative stress found in the current study in ruminants and in the above-mentioned studies in monogastric animals.

### Milk yield and main constituents

In our study, the HIO-fed cows showed higher milk yield when compared to the HPF-fed ones, while most of milk main constituents remained unaffected by diet. The only exception regarded milk urea content, which tended to be higher in the HIO-fed than HPF-fed cows.

Lauric acid generally exerts negative effects on milk production performance, such an effect being the consequence of reduced DMI by the cows. Faciola and Broderick [39] observed that lauric acid intakes from 0 to 438 g/cow/d linearly decreased milk yield as well as the contents and yields of milk fat, lactose and solids non-fat. In a subsequent trial, the same authors observed that lauric acid intakes of 288 g/cow/d depressed milk yield, 3.5% fat-corrected milk, as well as fat, protein and lactose yields, when compared to a control diet containing calcium soaps of palm oil FA [60]. These authors associated such response to depressed DMI (probably because of depressed palatability) and depressed fiber digestion with the diet containing lauric acid. However, it has to be remarked again that the lauric acid doses of the cited studies were much higher than that associated to dietary HIO in the present study. Differently from the results obtained in the above-mentioned studies, Nekrasov et al. [20] observed an increased (+ 8.0%) milk yield in dairy cows fed a 30:70 (F:C) diet (3.9 g/100 g DM of crude fat) supplemented with HIO at 100 g/cow/d, respectively, when compared to a control group of cows receiving an unsupplemented diet, with no significant changes in the contents of milk main constituents. Such results could be partly the consequence of an increased dietary energy supply, as it can also be hypothesized from the higher rumen total VFA concentration of the supplemented diet (10.37 vs. 6.56 mmol/100 mL), but a greater feed efficiency of the cows, as seen to have occurred in our trial, cannot be excluded; unfortunately, no information was provided about feed efficiency by these authors. In our study, we observed a 4.2% increase in milk production in the HIO-fed when compared to HPF-fed cows, and the experimental concentrates were formulated to be isoenergetic; therefore, the observed increase is most probably imputable to an increased feed efficiency [61], as later pointed out. Moreover, the observed significantly higher milk production in the HIO-fed cows in the current study may be also related to the improved antioxidant status of the cows. In fact, several studies associate the status of the immune system and cellular stress oxidation of the animals with variations in milk production and milk constituents (i.e., lower performance in the case of higher oxidative stress) [62]. We hypothesize that the observed increase in milk production and the higher resistance to oxidative stress in the HIO-fed cows in our study could be related to ghrelin stimulation by medium-chain SFA (and particularly lauric acid). Indeed, among its various physiological effects, ghrelin stimulates the release of the growth hormone (GH) from the pituitary gland through specific receptors in a dose-dependent manner [63]. During lactation, an enhanced release of GH in the bloodstream increases the blood flow to the mammary gland, consistently with an increased milk yield and with no effect on the synthesis of milk constituents [64].

As far as the observed tendency towards an increased milk urea content in HIO-fed cows is concerned, since no differences were observed for protein intake between treatments, a possible explanation could be attributed to the high lauric acid content in HIO by considering its known antimicrobial effect. Indeed, a shift in ruminal microbial communities, involving bacteria able to metabolize nitrogen, could have occurred. This hypothesis is supported by the recent findings of Vadroňová et al. [45], who found that the relative abundance of *Prevotellaceae* decreased in ruminal liquor of cows supplemented with medium-chain SFA when compared to cows supplemented with stearic acid. In the current study, the milk from both experimental groups showed low urea content [65], which is an expected result, consistent with the medium productivity of the Valdostana Red Pied breed. For this reason, the observed slight increase in the urea content of milk from HIO-fed cows should not be seen as a negative effect.

### Feed efficiency predictors

It is well known that HI, thanks to its lipid fraction rich in lauric acid, possesses antimicrobial and antifungal properties [17]. This may cause changes in ruminal microbial populations [66], have a direct impact on the animal’s nutrient utilization and, therefore, on its productive performance [67] and its overall feed efficiency [68]. Concerning this last-mentioned point, researchers and nutritionists have established different efficiency predictors, including the FCR and RFI [61]. The latter fits better to more complex conditions, as it happens in lactating animals. Indeed, our trial was conducted in a lactation stage commonly associated with large energy mobilizations of the body fat reserves and fluctuations of the energy balance of the animals, all factors that are not considered when using the FCR [69]. Thus, the observed inconsistencies in FCR and RFI results in the present study may derive from such discrepancies in the calculation of these efficiency predictors. Regarding the RFI, there are two major keys for the interpretation of the difference between groups. Efficient animals can be mainly recognized by (i) not showing any differences in the DMI but having a higher productive output; or (ii) by showing the same productive performance but having a lower amount of ingested feed when compared to the other group. The latter seems common in meat breeds and in Holstein cows [69, 70]. However, our results in Valdostana Red Pied cows agree with the first situation, which has mostly been described in minor dairy breeds (e.g., in Norwegian Red cows and in Assaf sheep) [71–73].

In the current study, both groups had the same input (feed intake); however, the HIO-fed group showed better milk performance, also when milk was corrected for energy (ECM) and for its fat and protein contents (FPCM), thus explaining the obtained higher efficiency. The observed higher milk production level in the HIO-fed group may also be the consequence of other factors, such as an improved state of the immune system of the animals (as outlined by the observed lower d-ROMs concentrations); indeed, the immune system is involved in the modulation and in the release of hormones involved in the development of the mammary gland (e.g., GH) [50].

Despite the small differences in RFI between groups, the higher feed efficiency observed for the HIO-fed cows is certainly a favorable result, which deserves further consideration in future trials addressed at evaluating the impact of HI-derived products in ruminant nutrition.

## Conclusions

Although insects are among the most promising novel feed ingredients to be used in animal nutrition thanks to their high sustainability and excellent nutritional profile, current research on their use in ruminant diets is limited and several knowledge gaps need to be covered. The results obtained in the current trial suggest that, at the proposed dose, HIO can be a viable alternative to HPF in lactating cow diets without compromising overall feed palatability, digestibility, and productive performance. Worth noting, HIO increased the feed efficiency of dairy cows by supporting a higher milk production together with an improved antioxidant status. This has several potential implications for dairy production. In fact, by incorporating HIO, dairy producers may improve feed efficiency, reduce reliance on traditional lipid sources with potential social and environmental concerns, improve Circular Economy models, and contribute to more sustainable feed production. Given the known antimicrobial properties of lauric acid, future efforts should be addressed to evaluate, in vivo, the effects of the dietary inclusion of HIO on rumen microbiota population, as well as fermentation and biohydrogenation patterns. Further studies should also be carried out to assess the effects of different dietary inclusion levels of HIO on the health and performance of dairy cows.

## Data Availability

The datasets analyzed in the current study are available from the corresponding author on request.

## Abbreviations

ADFom: Acid detergent fiber exclusive of residual insoluble ash
ADL: Acid detergent lignin
aNDFom: α-Amylase- and sodium sulfite-treated neutral detergent fiber exclusive of residual insoluble ash
BHB: β-Hydroxybutyrate
BW: Body weight
BWC: Body weight change
CP: Crude protein
DM: Dry matter
DMd: Total-tract dry matter digestibility
DMI: Dry matter intake
d-ROMs: Derivatives of reactive oxygen metabolites
ECM: Energy corrected milk
EE: Ether extract
EEd: Total-tract ether extract digestibility
FA: Fatty acid
FCR: Feed conversion ratio
FPCM: Fat and protein corrected milk
HI: *Hermetia illucens*
HIO: *Hermetia illucens* oil
HPF: Hydrogenated palm fat
NDFd: Total-tract neutral detergent fiber digestibility
NEFA: Non-esterified fatty acids
NSC: Non-structural carbohydrates
OM: Organic matter
OMd: Total-tract organic matter digestibility
RDP: Rumen degradable protein
RFI: Residual feed intake
ROS: Reactive oxygen species
RUP: Rumen undegradable protein
SAC: Serum antioxidant capacity
SCC: Somatic cell count
SEM: Standard error of the mean
SFA: Saturated fatty acids
TMR: Total mixed ration
uNDF: Undigested neutral detergent fiber
